# Insight into the seroepidemiology and dynamics of circulating serotypes of dengue virus over a 4 year period in western Uttar Pradesh, India

**DOI:** 10.1099/acmi.0.000567.v4

**Published:** 2023-06-23

**Authors:** Zeeshan Mustafa, Haris Manzoor Khan, Mohd Azam, Hiba Sami, Syed Ghazanfar Ali, Islam Ahmad, Adil Raza, Mohammad Azam Khan

**Affiliations:** ^1^​ Department of Microbiology, J.N. Medical College, Aligarh Muslim University, Aligarh, UP, India; ^2^​ College of Applied Medical Sciences, Al-Qassim University, Buraydah, Qassim KSA, Saudi Arabia; ^3^​ Viral Research & Diagnostic Laboratory, Department of Microbiology, J.N. Medical College, Aligarh Muslim University, Aligarh, UP, India; ^4^​ Department of Statistics & Operational Research, Aligarh Muslim University, Aligarh, UP, India

**Keywords:** dengue, epidemiology, PCR, seroprevalence, serotype

## Abstract

An important public health problem in India is dengue infection, with every year seeing an increase in cases of dengue fever. Dengue affects all individuals irrespective of their gender and age, although the infection rate is higher among males and younger people. Despite low severity in general, dengue virus can cause severe health conditions in some individuals. Genetic characterization of circulating endemic dengue virus (DENV) serotypes plays a significant role in providing epidemiological knowledge and subsequent vaccine development. In the present study, over a 4 year period, we assessed DENV transmission dynamics in major regions of western Uttar Pradesh in North India. ELISA tests were used to diagnose dengue, and PCRs were used to determine the circulating serotype. We found that dengue infection peaks after the rainy season and affects all sexes and ages. A total of 1277 individuals were found positive for dengue; among them, 61.7 % were male and 38.3 % were female. DEN-1 was found in 23.12 %, DEN-2 in 45 %, DEN-3 in 29.06 % and DEN-4 in 1.5 % of the dengue-infected individuals. All four DENV serotypes were circulating in the study area, and DENV serotype-2 (DEN-2) was the most prevalent serotype.

## Data Summary

No external data were used in the study. This article contains all supporting data, codes and protocols

**Impact Statement**

India is a developing country. Most states of India are subject to dengue outbreaks every year, making it a major cause of hospitalizations. Region-wise epidemiological studies on dengue virus transmission can help in controlling dengue spread. We perform a molecular epidemiological study on dengue-suspected patients in major districts of the western Uttar Pradesh region of North India; this will help in gaining a deeper understanding of dengue virus circulation by enhancing our knowledge of the regional distribution of dengue virus serotypes, which will help in effectively controlling the spread of dengue.

## Introduction

Dengue fever is the most prevalent viral infection of humans caused by dengue virus (DENV), which belongs to the family *Flaviviridae* of the genus *Flavivirus*; it is a single-stranded positive-sense RNA virus [1]. Female *Aedes* mosquitos are responsible for dengue transmission to humans [2]. A major proportion of the world’s population is at risk of contracting dengue fever, making it a serious public health concern [3]. Dengue is prevalent in tropical and subtropical regions, is endemic in most countries, and is most often reported in the Americas, Southeast Asia and the Western Pacific [4]. Despite its long history, dengue fever was first reported in India in 1956 in the Tamil Nadu district of Vellore [5]. Most states of India report dengue outbreaks every year, making it a major cause of hospitalizations [6]. Several factors contribute to the emergence of dengue fever, including ecological changes, climate change, epidemics of mosquito vectors and changing demographics [7]. Tropical and subtropical areas experience dengue infection throughout the year, and the disease is closely correlated with the rainy season, temperature, vector fluctuation and changing seasons [8]. Epidemics in different regions occur at different times. Peaks from non-endemic areas were determined based on the time at which imported cases appeared [9]. In Thailand and Myanmar, the peak time for dengue fever is during the monsoon season, which runs from May to October. In Vietnam, dengue fever cases are most common between June and December [10]. The peak season of dengue runs from February to May in Central and South American countries such as Brazil and from July to December in Puerto Rico [11]. In India, the peak season for dengue is the rainy season due to high humidity that leads to an abundance of breeding sites for mosquitos [12]. DEN-1, DEN-2, DEN-3 and DEN-4 are the four primary serotypes of DENV [13]. A new fifth type of DNV serotype-5 (DEN-5) was identified in the blood of patients in Sarawak, Malaysia, in 2007 [14]. Infection with any one of the four reported serotypes probably imparts lifetime immune protection to that particular serotype [15]. However, infection with a different serotype after primary infection with another serotype might lead to serious illness [16]. Several factors influence the severity of the second heterotypic dengue infection, including age, the time gap between the first and second infections, and serotype [17]. Heterotypic DENV infections pose a higher risk of fatal outcomes for young children because of their intrinsic risk of severe vascular permeability [18]. All four dengue serotypes are prevalent throughout India [19]. Several factors contribute to the clinical manifestations and severity of dengue infection, such as virus genetics, host characteristics and previous exposure to heterotypic dengue infections [20]. There are several clinical manifestations associated with DENV infection, including asymptomatic conditions, mild, non-specific fever, classical dengue fever (DF), and severe symptoms such as dengue haemorrhagic fever (DHF) and dengue shock syndrome (DSS) [21]. Traditionally, dengue fever is called ‘break-bone fever’ and causes symptoms such as fever, headache, myalgia, arthralgia, malaise and, in rare cases, severe dengue disease, characterized by plasma leakage, haemorrhage and death [22]. There is no specific treatment for DHF or DSS, although proper clinical diagnosis and management may control the effects of the disease [23]. There is currently no authorized treatment medication or vaccination for dengue. In some countries, however, only one live attenuated tetravalent dengue vaccine, CYD-TDV (Dengvaxia), has been approved [24, 25].

Diagnosis of dengue can be made in two ways: direct detection of viral antigen either by PCR/real-time PCR or by dengue NS1 antigen ELISA and indirect detection of IgM antibodies in blood plasma or serum [26]. DENV antigen can be detected in plasma or serum during the first week of infection, ideally during the period of fever [27]; during this period, viral RNA can be extracted for molecular diagnosis by PCR/real-time PCR using suitable primers [28].

Here, we perform a molecular epidemiological study on dengue-suspected patients in J.N. Medical College Hospital, AMU, Aligarh, India. As a result of this study, we will gain a deeper understanding of DENV circulation in Aligarh and adjacent districts, enhancing our knowledge of the regional distribution of DENV serotypes.

## Methods

### Study population and sample collection

This study was conducted at the Viral Research and Diagnostic Laboratory (DHR/ICMR), Department of Microbiology, Jawaharlal Nehru Medical College, AMU, Aligarh, UP, India, from January 2018 to December 2021. A total of 7256 blood samples collected from male and female patients of all ages with clinical features of dengue disease visiting J.N. Medical College Hospital were included in the study.

### Clinical samples

Venous blood samples of about 5 ml were collected from symptomatic patients in a sterile clot activator vacutainer, along with clinical and demographic information. After collection, samples were transported to the laboratory, maintaining a proper cold chain. The serum was separated by centrifugation and stored at −20 °C until further testing.

### Detection of dengue virus

#### ELISA

Two different ELISA kits were used for the detection of NS1Ag and IgM in all the samples:

1. ELISA assay for NS1Ag: dengue NS1Ag was detected using an InBios dengue NS1Ag ELISA kit according to the manufacturer’s instructions.
2. ELISA assay for IgM: dengue IgM antibodies were detected using an IgM MAC ELISA kit by NIV Pune as per the instructions provided.

### Serotyping of dengue

#### RNA extraction

A QIAamp Viral RNA Mini kit (Qiagen) was used to extract viral RNA from serum samples of dengue-positive patients, as per the manufacturer’s instructions.

#### cDNA synthesis

As per the manufacturer’s instructions, reverse transcriptase PCR was used to synthesize cDNA from the extracted RNA using the Thermo Scientific RevertAid First Strand cDNA Synthesis Kit.

#### PCR assay for serotyping

PCR was performed in 350 dengue NS1Ag ELISA-positive samples using a primer set defined by Lanciotti *et al.* [29] (Table 1). Briefly, we used 10 µl PCR master-mix 2× (Thermo Scientific), 1 µl forward primer (D1), 1 µl reverse primer (D2), 2 µl PCR-grade water and 6 µl cDNA; thermal-cycler conditions were 94 °C for 5 min, 94 °C for 30 s, 55 °C for 60 s and 72 °C for 120 s, 72 °C for 5 min and cooling at 4 °C. Serotypes DEN-1 to DEN-4 were detected by performing a second nested PCR using type-specific primers as defined by Lanciotti *et al.* (Table 1). The amplified product was then subjected to gel electrophoresis to analyse the result.

**Table 1.** Primer sequence and amplicon size for serotyping of DENV as defined by Lanciotti *et al*. [29]

Primer	Sequence	Amplicon size (bp)
Forward primer D1	5′-TCAATATGCTGAAACGCGCGAGAAACCG-3′	511
Reverse primer D2	5′-TTGCACCAACAGTCAATGTCTTCAGGTTC-3′	511
DEN-1 (TS1)	5′-CGTCTCAGTGATCCGGGGG-3′	482 (TS1 and D1)
DEN-2 (TS2)	5′-CGCCACAAGGGCCATGAACAG-3′	119 (TS2 and D1)
DEN-3 (TS3)	5′-TAACATCATCATGAGACAGAGC-3′	290 (TS3 and D1)
DEN-4 (TS4)	5′-CTCTGTTGTCTTAAACAAGAGA-3′	392 (TS4 and D1)

#### Real-time PCR assay

The detection of serotypes DEN-1 to DEN-4 was also performed by real-time PCR in a single step using LightMix Reflex Dengue Typing One-Step qRT-PCR kit (TIB MOLBIOL) following the manufacturer’s instructions.

#### Agarose gel electrophoresis

The amplified PCR product was then subjected to electrophoresis on a 1.5 % agarose gel, and a DNA ladder (80–1080 bp) was included in the run to compare the bands obtained during electrophoresis. After electrophoresis was completed, the agarose gel was visualized in the BioRad gel documentation system.

#### Statistical analysis

Statistical analysis was performed with SPSS (version 25). We performed a chi-square test to determine a significant association between dengue infection gender and the patient’s age. In every instance, a *P* value of ≤0.05 was used to assess the significance level.

## Results

In total, 7256 patients with suspected dengue infection from seven major districts of western UP (Aligarh, Hathras, Budaun, Kasganj, Bulandshahar, Etah and Sambhal) were tested between January 2018 and December 2021 (Table 2). Dengue was confirmed in 1277 patients (17.59%). Of these, 870 (68.12 %) samples were positive for dengue NS1Ag, 96 (7.51 %) samples for IgM, and 311 (24.35 %) samples for both NS1Ag and IgM. Among them, 788 (61.7 %) were male and 489 (38.3 %) were female patients. A large proportion of positive cases were from the age group of 11–20 years (33.12 %), followed by 21–30 years (30.38 %). Statistical analysis using the chi-square test revealed a significant association between dengue infection and gender and age (*P*<0.05) (Tables 2 and 3). From the results, we found that out of the total recruited dengue symptomatic patients, there were 5.1, 17.8, 10.5 and 19.4 % of male individuals and 3.7, 9.1, 7.4 and 11.7 % of female individuals infected by DENV during 2018, 2019, 2020 and 2021 respectively (Table 3) .

**Table 2.** Demographic profile of DENV-positive patients (%) (*n*=1277)

Year	Total dengue-positive (%)	Male (%)	Female (%)	DEN-1 (%)	DEN-2 (%)	DEN-3 (%)	DEN-4 (%)	*P*-value of dengue infection with gender
2018	8.85	58.1	41.9	16.6	33.3	50	0	0.004
2019	26.87	66.3	33.7	31.2	25.0	37.5	1.04	0.0001
2020	17.95	58.6	41.4	16.6	55.5	27.7	0	0.02
2021	31.07	62.5	37.5	23.0	65.3	8.6	1.9	0.0001

**Table 3.** Demographic profile of total patients screened for DENV (%) (*n*=7256)

Year	Males		Females		0–10 years		11–20 years		21–30 years		31–40 years		41–50 years		51–60 years		61–70 years		*P*-value of dengue infection with age
	+ve-	-ve	+ve	-ve	+ve	-ve	+ve	-ve	+ve	-ve	+ve	-ve	+ve	-ve	+ve	-ve	+ve	-ve	
2018	5.1	51.8	3.7	39.3	0.7	13.7	3	28.2	2.6	28.3	1.1	7.2	0.9	6.5	0.4	4.2	0.2	3	0.008
2019	17.8	39.1	9.1	34.0	2.3	3.5	9.5	24.8	7.8	23.5	2.7	7.7	2.2	5.6	1.2	3.9	2.7	2.6	0.0001
2020	10.5	53.1	7.4	29.0	1.2	7.7	6.1	24.9	5.4	25.6	2.2	9.2	1.6	5.8	0.7	4.6	0.4	4.5	0.04
2021	19.4	35.0	11.7	33.9	1.6	5.2	9.1	14.3	11.3	16.9	4.5	11.0	3.5	9.1	1.8	6.5	1.9	3.2	0.01

+ve, Positive; -ve, negative.

**Fig. 1.**
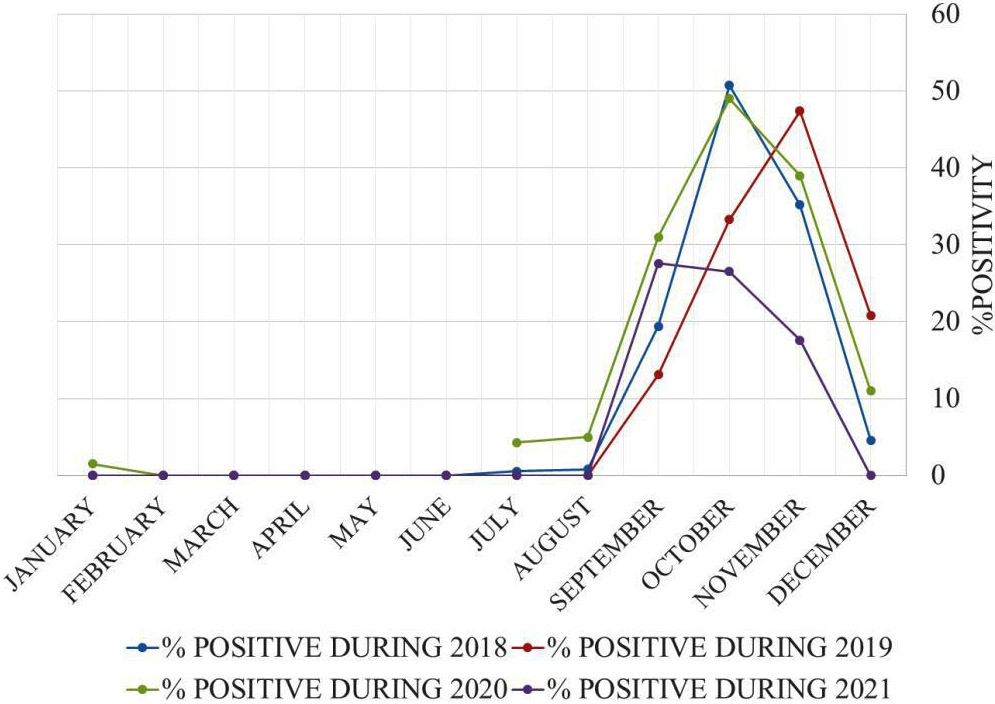
Seasonal distribution of dengue cases during 2018–21.

The seasonal distribution of the cases is given in Fig. 1. Rainfall peaks were associated with a higher frequency of recruited dengue patients (Fig. 1). In total, 99.8 % (1275/1277) of dengue-positive cases were detected from July to December.

**Fig. 2.**
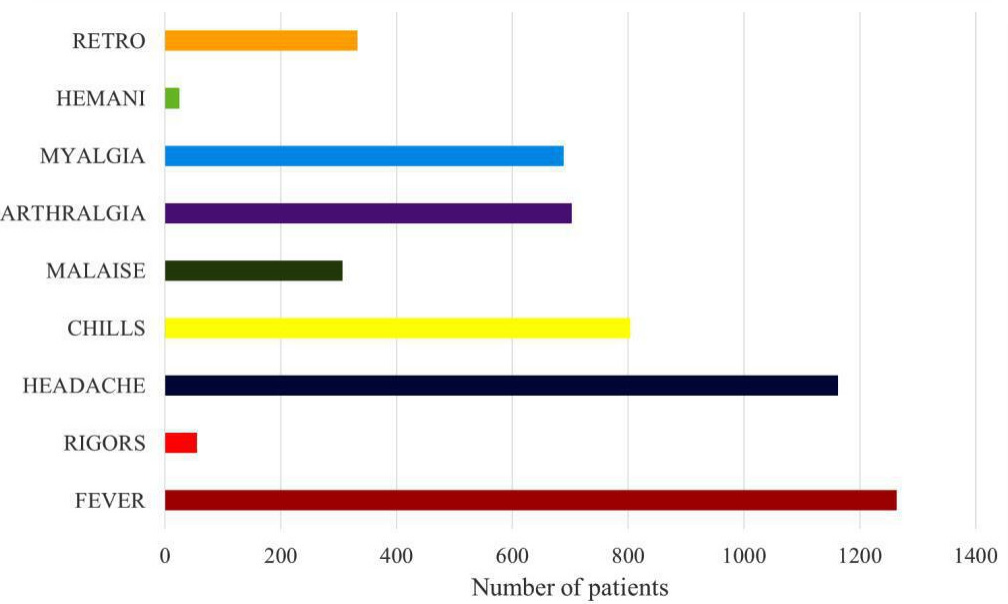
Clinical manifestation of dengue-positive cases (*n*=1277).

The proportion of positive dengue patients in 2018, 2019, 2020 and 2021 was 8.85, 26.86, 16.96 and 31.07 %, respectively (Table 2). Major symptoms reported by DENV-positive patients in our study were fever (99 %), headache (91 %), chills (63 %), arthralgia (55 %), myalgia (54 %) and retro-orbital pain (26 %) (Fig. 2).

**Fig. 3.**
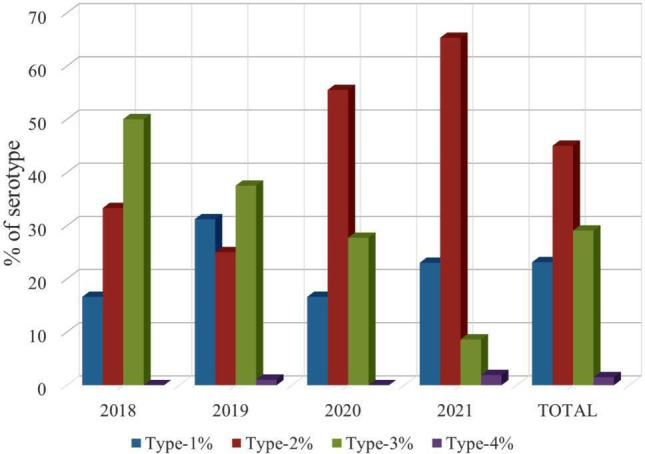
Dengue serotype distribution between 2018 and 2021 in western Uttar Pradesh.

A total of 320 samples gave positive results with the PCR. The distribution of serotypes was as follows: DEN-1 in 23.12 % of positive patients, DEN-2 in 45 %, DEN-3 in 29.06 % and DEN-4 in 1.5 %. DEN-3 was the predominant serotype during 2018 and 2019 (50 and 37.5 %, respectively), followed by DEN-2 (33.3 and 25 %, respectively), whereas during 2020 and 2021, the DEN-2 serotype was predominant (55.5 and 65.3 % respectively) followed by DEN-3. Serotype DEN-1 was also detected each year, but serotype DEN-4 was detected only in 2019 and 2021 (Fig. 3). Two patients were positive for mixed serotypes: DEN-1 and -4 in 2018; and three patients were positive for DEN-2 and -3 in 2019.

## Discussion

This study analyses the seroprevalence and epidemiology of 4 years of dengue circulation in seven districts (Aligarh, Hathras, Budaun, Bulandshahar, Kasganj, Etah and Sambhal) of the western Uttar Pradesh region of North India. In the Aligarh region, the climate is typically subtropical humid, influenced by the monsoon, which begins in late June and lasts until September [30]. DENV infection spreads through insect vectors *Aedes aegypti* and *Aedes albopictus*; these mosquitos breed in clean and clear stagnant water in hot and humid climates [31]. Our study found that dengue is most prevalent from September to November. In 2018 and 2020, the dengue peak occurred in October; in 2019, the peak occurred in November; and in 2021, the peak occurred in September. Various studies from Delhi, Rajasthan and Hindu Kush Himalayan Region have also reported that dengue is most prevalent from September to November [32–35]. These results suggest that dengue infection spread depends on mosquito vectors; therefore, controlling mosquito breeding is crucial in preventing DENV spread, especially during the rainy season. Also, water should not be allowed to accumulate in dark places such as containers, boxes, vehicle tires, etc., which play an important role in providing sites for mosquito breeding. The findings from our study for the period January 2018 to December 2021 reveal that dengue-positive cases were highest in 2021 (31.07 %), followed by 26.86 % in 2019, 20.15 % in 2020 and 8.85 % in 2018. The National Center for Vector Borne Diseases Control (NCVBDC) also shows similar data for dengue infection in Uttar Pradesh [36]. More dengue-positive cases were found in males (61.7 %) compared to females (38.3 %). Several other studies from different regions such as Malaysia, Western Uttar Pradesh and other regions of India have also reported that males are more prone to dengue infection than females [37–40]. In our study, we found that a large proportion of dengue-positive patients were in the age range 11–20 years (33.12 %), followed by 21–30 years (30.38 %). The higher number of dengue-positive infections among individuals in the age group 11–20 years may be due to local movement of these individuals; for example those in the age group 11–20 years are generally at school or college and play outside during the day and thus are easily bitten by the dengue virus vector *Aedes* mosquitoes, which mainly bite during daytime hours [41]. Similar studies from Delhi, West Bengal, Odisha and Central India also found that dengue infection is most common among individuals in the age group 11–20 years, followed by 21–30 years [42–45]. Therefore, public awareness about the spread of dengue infection should be raised to prevent the infection. The use of mosquito repellents is recommended, especially for young children; also, everyone should follow guidelines to help prevent mosquito breeding by disposing of waste properly and blocking rainwater accumulation in waste containers, tyres, bags, etc. In the present study, fever and headache were reported by the majority of dengue-infected patients, followed by chills, arthralgia, myalgia and retro-orbital pain. Previous studies in Delhi, Assam, Uttar Pradesh and West Bengal also reported similar symptoms in dengue-infected patients [46–49].

All four DENV serotypes are reported in India [50]. Various dengue outbreaks in Uttar Pradesh also show the circulation of all four DENV serotypes. One study from 2009 to 2012 in Uttar Pradesh found that DENV serotype-2 was the most prevalent serotype circulating in Uttar Pradesh, followed by serotype-3 and then serotype-1; they did not find serotype-4 at all [51]. Other studies in Delhi, North India, during 2013, 2014 and 2015 reported serotype-2 as the major circulating DENV serotype, followed by serotype-1 [52–54]. In contrast, another study during the 2014 dengue outbreak in New Delhi reported serotype-1 as the major circulating serotype, followed by serotype-2 [55]. On the other hand, one study during the 2016 and 2017 dengue outbreak in New Delhi reported DENV serotype-3 as the most prevalent circulating serotype in the region followed by serotype-2 [56, 57].

In the present study from 2018 to 2021 in Uttar Pradesh, we found that dengue serotype-2 (DEN-2) was the most prevalent serotype circulating in Aligarh and adjacent districts, followed by serotype-3 (DEN-3) and serotype-1 (DEN-1). Serotype-4 (DEN-4) was detected only in 2019 and 2021; very few patients were found positive for mixed serotypes: two patients of DEN-1 and -4 in 2019 and three patients of DEN-2 and -3 in 2019 in the Aligarh region; however, none of the patients with mixed infection with one or more serotypes were reported to develop severe dengue. During the years 2018 and 2019, serotype-3 (DEN-3) (50–37.5 %) was the most prevalent serotype circulating in Aligarh and adjacent regions, followed by serotype-2 (DEN-2) (33.3 and 25 %). By contrast, during 2020 and 2021, DENV serotype-2 (DEN-2) (55.5 and 65.3 %) was the most prevalent serotype, followed by serotype-3 (DEN-3) (27.7 and 8.6 %). One study from Lucknow, Uttar Pradesh, during 2011 and 2013 reported DEN-3 as the most prevalent circulating serotype followed by DEN-1 [58]. Another study during 2018 also reported DEN-3 as the most prevalent serotype but this time followed by DEN-2; they also detected dengue serotype-4 (DEN-4) for the first time in their study [59]. The results from our study and various other studies suggest that, at regular intervals, the prevalent serotype of DENV has changed; until 2016, dengue serotype-2 (DEN-2) was the major circulating serotype, and then in 2016, 2017 and 2018, serotype-3 (DEN-3) took over and became the major circulating serotype. During this period, dengue serotype-4 (DEN-4) was also detected but with very few cases, and again in 2020 and 2021, serotype-2 (DEN-2) became the most prevalent serotype of DENV circulating in Uttar Pradesh. From the present study, we found that dengue serotype-2 (DEN-2) spread rapidly and caused a large number of infections, as the highest number of dengue infections was found during the 2021 dengue outbreak, in which serotype-2 (DEN-2) was the most prevalent serotype, but the number of dengue cases was lowest during the 2018 dengue outbreak in which serotype-3 (DEN-3) was the most prevalent serotype. DENV serotype-2 is better adapted to transmission by mosquitoes [60]. One study from 1990 to 2015 showed that DENV serotype-2 (DEN-2) was responsible for causing a large number of dengue outbreaks around the globe [61]. Therefore, regular monitoring of prevalent serotypes would help control spread of the disease.

One of the limitations of our study was that we do not have data on the mortality of dengue-infected patients; hence we could not identify which serotype is responsible for higher numbers of dengue-related mortalities. We were also unable to determine the prior infection history of those with secondary dengue infection. Despite these limitations, our study will provide insight into the seroprevalence pattern of DENV, which changes from time to time; this will open the way to further research on how these serotypes behave and spread and the reason behind the shift from one prevalent serotype to another.

## Conclusion

In recent years, dengue epidemiology studies have noted significant variation in DENV serotypes across India. The present study concludes that all four DENV serotypes circulate in Aligarh and adjacent districts of western Uttar Pradesh in North India. Serotype-2 (DEN-2) is the most prevalent followed by serotype-3 (DEN-3). Gender and age are significantly associated with dengue infection. Male individuals are more prone to infection than females, and individuals in the age group 11–30 years are at high risk of getting the infection.
